# State of Lunacy in England

**Published:** 1859-10-01

**Authors:** 


### Art. VIII.—STATE OF LUNACY IN ENGLAND.

The Thirteenth Report of the Commissioners in Lunacy, recently published, is of considerable interest, notwithstanding that it follows so rapidly upon, and has had several of its details anticipated by, evidence published in the report of the Select Committee on Lunatics which sat during the late Parliament. The contents of the Commissioners' Report do not well admit of being abstracted, but we shall endeavour to indicate some of the more important particulars given.

Since the 31st March, 1858, three of the public Asylums announced last year as in course of erection have been opened, to wit, those for Durham, Cambridge, and Northumberland. The United Asylum for the county and borough of Cambridge, and the Isle of Ely, is situated about 3½ miles from Cambridge, and it "is built upon a site moderately elevated, commanding extensive views. The land consists of about 57 acres; the surface is fine loam on a chalk subsoil. The building is designed to accommodate 250 patients." (p. 5.) The Asylum for the county of Durham is situated at Sedgefield, about 11 miles from Durham, and is estimated to accommodate 312 patients—157 males and 155 females.

"The cost of erection of the Asylum was 31,480*l.*; viz.—land, 52 acres, 4000*l.*, and buildings and fittings, 27,480*l.* The total average cost per patient being thus about 100*l.*

"The Asylum consists of a main building of three stories, and two separate blocks of two stories each, in connexion respectively with the workshops and laundry, and containing day-rooms on the ground, and associated dormitories on the upper floor.

"In the centre of the main building, the principal approach to which is from the north, are the superintendent's residence and the general dining-hall, over which is the Chapel. The male and female patients occupy respectively the western and eastern divisions of the Asylum.

"The second floor of the main building is occupied only at night.

"According to the original design there were on the second floor in each division three dormitories opening into a passage towards the north. Upon further consideration the partition walls were omitted, and the upper story on each side was converted into a large dormitory containing 50 beds, and warmed by open fires.

"The several wards are heated by open fires only. The dining-hall and Chapel are warmed by a hot-water apparatus."—(p. 12.)

The Commissioners report favourably of the condition of the patients already admitted into both asylums, and of the arrangements; and they also make several minor suggestions to promote the efficiency of the institutions.

With one or two exceptions the condition of the public asylums, and the patients contained in them, throughout the kingdom, is very favourable; and the details given by the Commissioners in respect of each asylum, give a most satisfactory impression as to the persistent efforts of the different asylum authorities to keep up or improve the efficiency of the establishments. Moreover, the suggestions of the Commissioners show that not even the veriest minutiæ of management and arrangement escape their eyes.

The Haverfordwest asylum, however, is still characterized by glaring unfitness and mismanagement. In our notice of the Twelfth Report of the Commissioners, we pointed out how this asylum had set the Commissioners at defiance since 1854, and how towards the end of 1857 the interference of the Secretary of State was requested, and the medical officer was superseded. Little benefit, it would now appear, has resulted from this change, and the asylum still retains its discreditable character. The West-Riding of York Asylum is exempted from a favourable notice by a high rate of mortality, and many defects in the internal arrangements and management. How needless this is, and we hope that it may now be said was, is made manifest by a note appended to the Commissioners' Report, which tells us, that since the comparatively recent appointment of Mr. Cleaton, as medical superintendent of the asylum, the general condition and management "have been reported as materially improved." (p. 41.)

Certain remarks of the Commissioners on the medical staff of the huge, overgrown, yet still increasing Colney Hatch Asylum, are well worthy of attention.

"The medical staff at this large asylum at present consists only of two medical superintendents, each of whom has an assistant, who acts as dispenser. These gentlemen have the entire medical and moral control and care of 1285 patients. It is manifest that anything like individual treatment must be limited to a very small proportion of the cases, and we fear that, with the mass of the patients, the superintendents must necessarily depend mainly upon the good conduct and trustworthiness of the attendants. Moreover, the chance of cure must, as we apprehend, be greatly reduced; such chance being still further diminished by the fact that, during the last six months, there has been no medical assistant on the male side."

Only one or two examples of restraint, and those of the slightest character, are reported from the different asylums.

The Commissioners again urge the necessity which exists for additional asylum accommodation, and on the most eligible modes of making such provision they have the following remarks.

"The mode by which additions have been made to county asylums varies in different counties. As a rule, we have suggested the erection of detached buildings of a simple and inexpensive character, rather than additions to the main structure, on the ground that additions must generally partake of the character of the original building, and thus often entail the necessity of erecting new wards of too expensive a construction. Such wards are, as we think, quite unnecessary for many of the chronic and idiotic cases which accumulate in all large asylums, and are not required for those patients who can be regularly employed in active occupations. Above all, we have invariably found that patients removed from the long galleries of an asylum, to the more home-like apartments of a detached building, have not only presented a more cheerful and comfortable appearance, but have themselves expressed their satisfaction at the change."

These suggestions cannot be too highly commended.

A progressive improvement in the condition and management of the metropolitan licensed houses is reported, and it is stated that "the entries made by the Commissioners at the several provincial houses are for the most part favourable. Four houses have been closed in the provincial districts during the year, and one new license granted."

The Commissioners make known the circumstances which guide them in granting licenses, and they urge that it is important that they should have legal power to demand from the proprietors of licensed houses accounts of the payments made for, and amounts expended upon, patients. Thus, it is evident that the offensive and unjustifiable imputations respecting the probity of proprietors of licensed houses, which were made by the Earl of Shaftesbury in his evidence before the Select Committee on Lunatics, and upon which he based a similar proposition, are indulged also by the other Commissioners in Lunacy. We treated of this matter in our last number, but we may repeat here a remark we then made concerning the 26th clause of the proposed Bill to "Amend the Law concerning the Care and Treatment of Lunatics," which was submitted to the Select Committee, and which provided that the proprietors of licensed houses, and persons having charge of single patients, should furnish information to the Commissioners as to payment for patients:—

"This clause does not provide that due and sufficient cause being shown, warranting the suspicion of neglect of a patient, the Commissioners shall have the power of requiring an account to be submitted to them of the receipts from and expenditure for the patient, but gives them an unlimited power of requiring such accounts to be laid before them, when, how, and for whatever cause they might think fit! Such a proposition as this evidently emanates from the same suspicious spirit which infects the whole of Lord Shaftesbury's evidence respecting medical men practising in lunacy. It is not a *bonâ fide* provision, and would no doubt, if it were enacted, have the same fate as all vexatious legislative provisions; but in the meantime, it, and the rest of the clauses of the bill conceived in the same spirit, would have a diametrically opposite effect to that which they were intended to have."—p. 407.

x what would it be if removed in a home-like family house.

The great importance of having competent attendants in an asylum, the value of night attendants, the signification to be attached to the word "seclusion" in the Lunacy Act, and the indispensableness of a good, nutritious diet among pauper lunatics, are discussed by the Commissioners. They have issued a useful circular concerning the qualification of attendants; and they hold that "any amount of compulsory isolation in the day-time, whereby a patient is confined in a room, and separated from all associates, should be considered as seclusion, and recorded accordingly."

Certain remarks concerning the property of lunatics deserve consideration:—

"We avail ourselves of this occasion urgently to press upon your Lordship's attention, with a view to early legislation, the great hardship and injustice entailed upon a large number of the insane and their families by the present dilatory and expensive provisions of the law for the administration of the property and income of insane persons of very limited means, more especially those whose mental malady is of a temporary, and probably curable character.

"One of the objects of the 'Lunacy Regulation Act, 1853,' was to provide a remedy for this great evil. The result of that enactment, in this respect, has entirely disappointed public expectation. The 120th section, which was specially designed to meet the cases referred to, has proved practically inoperative, by reason of the large and ruinous expense attending the necessary proceedings. We are informed by the Registrar in Lunacy that in no case can the requisite authority to represent the lunatic be obtained at a less cost than 75*l*. The provision is therefore illusory, and inapplicable as respects that large class, peculiarly objects of compassion, whose families are, as a first result of the disorder which has afflicted themselves, overwhelmed with misery, and frequently reduced to pauperism.

"These observations have especial reference to persons of limited life incomes, and to small tradesmen; and, as a striking illustration of our views, to the cases of poor governesses, whose anxious calling often induces temporary insanity, and who may have accumulated savings to a trifling amount, and prudently invested them in the funds.

"We would further observe, that legislation is required not only with reference to the interests of lunatics and their families, but for the protection of public companies, tenants, and others, owing dividends, rents, and other debts, and ready and desirous to pay them on receiving a legal discharge.

"We may mention, among many others which have been brought under our notice, the case of a Fire Insurance Company indebted to a lunatic upon a policy, and unable, without circuitous, inconvenient, and expensive proceedings, to relieve themselves from their liability.

"It is not within our province to indicate the mode by which the property and income of the class of the insane to whom we have adverted could best be rendered available for their benefit and that of their families, but we deem it our duty strongly to express our opinion that, in all cases such as those under consideration, legal provision of an expeditious and inexpensive kind ought to be made for investing some person with authority to act for the Lunatic, as civilly dead. We venture further to submit that, unless and until a relative or friend be found, competent and willing to act in that capacity, the duty should devolve upon a public officer, in the nature of an official committee."

We commend to notice the formation of a fund by the Commissioners, for the relief of those pauper lunatics who, "as repeatedly happens," when entitled to a discharge from an asylum, have neither friend nor workhouse to receive them. A donation of 300*l.*, and subscriptions to the amount of 100*l.* by the Commissioners and their Secretary, form the nucleus of this fund, which is entirely a matter of voluntary contribution. The Colney Hatch and Hanwell Asylums have special funds for the purpose which have worked most beneficially.

The state of single patients is considered by the Commissioners, and, foiled in their efforts to carry out as rapidly as they could wish, improvements which they deem to be necessary in the management and control of such patients, as well as to perform a sufficiently extensive visitation by which unjust and improper treatment would be prevented, the Commissioners express the following opinion:—

"It is our conviction that the law will continue to be extensively violated or evaded, as respects this most helpless and neglected class of the insane, until medical practitioners are required, under a severe penalty, to give notice to this office of the names and residences of all persons who shall, for a given period, have been professionally attended to by them, as insane patients. We believe that such an enactment would produce numerous disclosures, which, in the absence of such a legal provision, medical practitioners are reluctant to volunteer."—(p. 78.)

We feel assured that the proposition implied in this opinion will prove most offensive to the medical profession, and that portion of the public next to them whom it most concerns. Nay, it is tolerably certain that, if the Commissioners' suggestion be acted on, a piece of vexatious and inutile legislation will be the result.

Can anything be more obnoxious to the feelings than the notion that the medical man should, in reference to lunacy, be transformed into a compulsory informer? That the bond of confidence which exists between the patient and his friends or relatives and the doctor in the earlier stages of lunacy is to be at once and abruptly broken? That the many reasons which, at the outset, may induce a family to wish that threatened, or manifest, and perhaps temporary lunacy in a member of the family, may be kept private, and into which reasons we have no right to pry, should by an arbitrary legal enactment be made of no avail; and that families are to be made distressingly aware that no privacy can be maintained unless the *medical man be kept out of the house!* That the fact of lunacy may subject a household to legal inspection! Think of the neglect in early attendance, and of the quackery that such a state of things would induce.

But apart from these objections, which are not to be lightly unheeded, there are others, and if anything more formidable ones. Granting (for the sake of argument) that the condition of single patients is, in many instances, as unsatisfactory as the Commissioners state, we entirely disbelieve that the method in which they seek to overcome the evil is the right one; nay, more, we assert that it is manifestly erroneous. The law already makes it a misdemeanour for any person to receive a lunatic into his house for profit, without making known the same to the Commissioners, and without a certificate of lunacy; and it calls upon every medical man attending upon such a patient to report upon the condition of the said patient once annually to the Commissioners. We have here evidently the germ of the Commissioners' recent suggestion, and it is equally evident that the medical man has not hitherto considered himself warranted in so entirely ignoring the wishes of the patient or his friends as to fulfil the expectations of the statute. Is there any probability that the threat of fine would in anywise alter his conduct in this respect? The Commissioners have found that the police functions assigned to the doctor by the Act have not been carried out; who is to exercise police functions upon *him*, if the suggestion of the Commissioners should pass into law? Unless the Commissioners propose to give part of the penalty to the informer, we see no chance of the success of such an enactment, except as a source of irritation. Moreover, will the families who may have the misfortune to have insane members, or a tendency to insanity among them, consent to so arbitrary an interference with a question of private judgment? We fancy not.

Rest assured that the Sangrado principle applied to lunacy will never act. Law, and yet again law, and still more law, is an empirical method of procedure which can lead to no good. Lunatics are already sufficiently well larded with law. What, then, is wanted? First, a much better state of feeling respecting lunacy and lunatics among the people in general, than now exists. But how is this to be brought about if the Commissioners persist in speaking of and legislating respecting lunacy, as if medical men, in reference to it, were simply ogres? The confidence between the medical profession and the Commissioners is weakened on the one hand, and between the public and medical men on the other, and the unhappy patient—the incipient lunatic, or single patient, as a necessary result, is victimized. Friends are frightened of calling in the medical man, in the first place—frightened of the asylum, in the second place—frightened of the certificate of lunacy, in the third place—and even the, to the public, mysterious Lunacy Commissioners, known only by trenchant Acts of Parliament, assume a dreadful and portentous character.

How unfitted the law is to deal with the intricate workings of lunacy matters, is well shown in an instance contained in the present Report of the Commissioners. They tell us of a licensed house existing in the kingdom, conducted on principles diametrically opposed to those now received as the right ones. Why do not the Commissioners close it, then?

"In this case we have reason to believe that no intentional harshness or neglect has occurred on the part of the proprietor, *who doubtless considers himself justified* in pursuing a course of treatment, and in providing a species of accommodation, which forms an exception to those of all similar establishments in the kingdom. *Had it not been for this belief on our part*, we should have thought it our duty some time ago to *take steps to prevent the renewal of licence*."—(p. 52.)

And yet the Commissioners, who can thus admit the private opinion of an individual, *in his own interest*, to outweigh the written, specified law, would enact a law to compel men to entirely set aside their own private judgment and the judgment of others!

Let the Commissioners seek to obtain the confidence of the medical profession, and to spread a just knowledge of the requirements of the insane in the kingdom, and we have no doubt that they will much more readily and satisfactorily attain their aim, in respect to single patients, and the welfare of private lunatics generally, than by vexatious legal enactments. The great difficulty in the way of exercising a satisfactory control over single patients, is the feeling of the friends; and this will only be overcome by kindly remonstrances and watchfulness, not by compulsion; and we believe that the Commissioners underrate their own powers in wishing to have additional legislation upon private patients. Indeed, from the instance we have quoted, and we could give other instances, it is evident the Commissioners have more law than they can well make use of. We know how steadily improvements are going on in the treatment of the insane, under the watchful care of the Board. Doubtless it may have been disheartened by difficulties, but we have infinitely more confidence in those difficulties being overcome by vigilant care than by legislation. We have spoken thus strongly of two measures suggested by the Commissioners, in their Report; for in the present state of things we estimate the persistent, individual, and collective action of those gentlemen at a much higher rate than that of additional enactments.

The Report terminates by quoting a legal opinion that lunatics, declared so by inquisition, and placed in the position of single patients, are to be treated as such; with some remarks on the propriety of ensuring the visits of friends to lunatics in asylums, and an account of the difficulties which still impede the erection of an asylum for the city of London.

The appendix of the Report contains two series of statistical tables, the one referring to patients in asylums, the other to pauper lunatics and idiots.

The first tables are arranged upon a better and more comprehensive plan than in previous Reports, and we trust are to be taken as an indication that the Commissioners are about to place the statistics of insanity in good and proper order; but it is to be regretted that the tables are printed in a scrawling, ungainly fashion.

On the following page is the summary of the tables, and we have added the totals for the previous year.

These figures show a total increase of 601 patients admitted during the year 1858, and an increase of 350 pauper lunatics as compared with 915 during 1857.

The abstract of annual returns of pauper lunatics and idiots belonging to the several unions in England and Wales, on the 1st January, 1859, gives the following results:—

| | Number of Patients. | In County or Borough Asylums. | In Registered Hospitals, or Licensed Houses. | In Workhouses. | In Lodgings, or Boarded Out. | Residing with Relatives |
|---|---|---|---|---|---|---|
| England. | 28,104 | 14,194 | 1,922 | 7,410 | 767 | 3,811 |
| Wales.... | 1,754 | 359 | 175 | 232 | 345 | 643 |
| Total..... | 29,858 | 14,553 | 2,097 | 7,642 | 1,112 | 4,454 |

Returns from the Poor-Law Commissioners, published in previous numbers of the Lunacy Commissioners' Reports, give as the number of pauper lunatics and idiots chargeable to parishes in 1847 and 1857, the following figures:—

1847—18,065

1857—27,693

1859—29,858

Much of the increase in the foregoing numbers is due to greater care in recording cases of pauper lunacy.

### SUMMARY.

| | Number of Patients 1st Jan. 1858. | | | | | | | Admissions during the Year 1858. | | | Discharges during the Year 1858. | | | | | |
|---|---|---|---|---|---|---|---|---|---|---|---|---|---|---|---|---|
| — | Private. | | | Pauper. | | | Total Lunatics. | | | | Total Number. | | | Number Recovered. | | |
| | M. | F. | Total. | M. | F. | Total. | | M. | F. | Total. | M. | F. | Total. | M. | F. | Total. |
| County and Borough Asylums. | 134 | 97 | 231 | 6777 | 8112 | 14,889 | 15,120 | 2413 | 2572 | 4985 | 1187 | 1427 | 2614 | 887 | 1078 | 1965 |
| Hospitals ............ | 762 | 730 | 1492 | 95 | 79 | 174 | 1666 | 431 | 473 | 904 | 253 | 354 | 607 | 134 | 207 | 341 |
| Metropolitan Licensed Houses. | 675 | 630 | 1305 | 491 | 827 | 1318 | 2623 | 561 | 605 | 1166 | 422 | 494 | 916 | 157 | 203 | 360 |
| Provincial Licensed Houses | 719 | 720 | 1439 | 550 | 489 | 1039 | 2478 | 598 | 454 | 1052 | 393 | 397 | 790 | 208 | 192 | 400 |
| | 2290 | 2177 | 4467 | 7913 | 9507 | 17,420 | 21,887 | 4003 | 4104 | 8107 | 2255 | 2672 | 4927 | 1386 | 1680 | 3066 |
| Royal Naval hospital ............ | 126 | — | 126 | — | — | — | 126 | 39 | — | 39 | 15 | — | 15 | 13 | — | 13 |
| | 2416 | 2177 | 4593 | 7913 | 9507 | 17,420 | 22,013 | 4042 | 4104 | 8146 | 2270 | 2672 | 4942 | 1399 | 1680 | 3079 |
| 1st Jany. 1857....... | Males...9962 | | | Females...11275 | | | 21,237 | 3895 | 3999 | 7895 | 2279 | 2588 | 5197 | 1299 | 1566 | 2865 |

| | Deaths during the Year 1858. | | | | | | | | | | | | | | |
|---|---|---|---|---|---|---|---|---|---|---|---|---|---|---|---|
| | Total Number. | | | From Suicide. | | | | | | From Accidents or Violence. | | | | | |
| — | | | | Act committed in Asylum. | | | Act committed before Admission. | | | Incurred in Asylum. | | | Incurred before Admission. | | |
| | M. | F. | Total. | M. | F. | Tot. | M. | F. | Tot. | M. | F. | Tot. | M. | F. | T. |
| County and Borough Asylums. | 888 | 761 | 1649 | 1 | 3 | 4 | — | — | — | 6 | 2 | 8 | — | — | — |
| Hospitals ............ | 64 | 41 | 105 | — | 3 | 3 | — | — | — | 1 | — | 1 | — | — | — |
| Metropolitan Licensed Houses. | 178 | 144 | 322 | 1 | — | 1 | — | — | — | 3 | 1 | 4 | — | — | — |
| Provincial Licensed Houses | 131 | 86 | 217 | 3 | 1 | 4 | — | — | — | — | 4 | 4 | — | — | — |
| | 1261 | 1032 | 2293 | 5 | 7 | 12 | — | — | — | 10 | 7 | 17 | — | — | — |
| Royal Naval hospital ............ | 13 | — | 13 | — | — | — | — | — | — | — | — | — | — | — | — |
| | 1274 | 1032 | 2306 | 5 | 7 | 12 | — | — | — | 10 | 7 | 17 | — | — | — |
| 1st Jany. 1857....... | 1231 | 926 | 2168 | 11 | 3 | 14 | 1 | — | 1 | 9 | 3 | 12 | — | — | — |

### PATIENTS REMAINING 1ST JANUARY, 1859.

| — | Private. | | | Pauper. | | | Total Lunatics. | Number deemed Curable. | | | Found Lunatic by Inquisition. | | | Criminals. | | | Chargeable to Counties or Boroughs. | | |
|---|---|---|---|---|---|---|---|---|---|---|---|---|---|---|---|---|---|---|---|
| | M. | F. | Total. | M. | F. | Total. | | M. | F. | Total. | M. | F. | Total. | M. | F. | Total. | M. | F. | Total. |
| County and Borough Asylums.................... | 122 | 105 | 227 | 7127 | 8488 | 15,615 | 15,842 | 879 | 794 | 1673 | 8 | 3 | 11 | 242 | 84 | 326 | 644 | 710 | 1354 |
| Hospitals .............................................. | 874 | 766 | 1640 | 109 | 109 | 218 | 1858 | 137 | 202 | 339 | 22 | 12 | 34 | 122 | 18 | 140 | — | — | — |
| Metropolitan Licensed Houses.................... | 663 | 624 | 1287 | 464 | 800 | 1264 | 2551 | 140 | 191 | 331 | 69 | 63 | 132 | 31 | 5 | 36 | 35 | 84 | 119 |
| Provincial Licensed Houses........................ | 862 | 736 | 1598 | 469 | 456 | 925 | 2523 | 209 | 204 | 413 | 79 | 44 | 123 | 137 | 43 | 180 | 70 | 109 | 179 |
| | 2521 | 2231 | 4752 | 8169 | 9853 | 18,022 | 22,774 | 1365 | 1391 | 2756 | 178 | 122 | 300 | 532 | 150 | 682 | 749 | 903 | 1652 |
| Royal Naval Hospital.................................. | 137 | — | 137 | — | — | — | 137 | 17 | — | 17 | — | — | — | — | — | — | — | — | — |
| | 2658 | 2231 | 4889 | 8169 | 9853 | 18,022 | 22,911 | 1382 | 1391 | 2773 | 178 | 122 | 300 | 532 | 150 | 682 | 749 | 903 | 1652 |
| | | | | | | | | Included in Total Lunatics. | | | | | | | | | | | |
| Patients remaining 1st January, 1858 ......... | 2508 | 2230 | 4738 | 7985 | 9587 | 17,572 | 22,310 | 1255 | 1528 | 2783 | 171 | 124 | 295 | 490 | 143 | 633 | 694 | 796 | 1490 |

